# Liquid–liquid extraction solvent selection for comparing illegal drugs in whole blood and dried blood spot with LC–MS–MS

**DOI:** 10.1093/jat/bkae081

**Published:** 2024-10-05

**Authors:** Melike Aydoğdu, Hasan Ertaş, Fatma Nil Ertaş, Serap Annette Akgür

**Affiliations:** Institute on Drug Abuse, Toxicology and Pharmaceutical Science, Ege University, Bornova, Izmir 35100, Türkiye; Chemistry of Department, Faculty of Science, Ege University, Bornova, Izmir 35100, Türkiye; Chemistry of Department, Faculty of Science, Ege University, Bornova, Izmir 35100, Türkiye; Institute on Drug Abuse, Toxicology and Pharmaceutical Science, Ege University, Bornova, Izmir 35100, Türkiye

## Abstract

This study focused on the simultaneous detection of amphetamine, 3,4-methylenedioxy methamphetamine, morphine, benzoylecgonine, and 11-nor-9-carboxy-tetrahydrocannabinol in whole blood and dried blood spot (DBS). It is aimed to select a solvent mixture for liquid–liquid extraction technique employing liquid chromatography–tandem mass spectrometry (LC–MS-MS). The obtained DBS results were compared with the whole blood samples results. A simple, rapid, and reliable LC–MS-MS method was developed and validated for all analytes in whole blood and DBS. LC was performed on a Hypersil Gold C18 column with an initial gradient of 0.01% formic acid, 5 mM ammonium format buffer in water, and acetonitrile at 0.3 ml/min with 7.5 min runtime. A methanol:acetonitrile (40:60 v/v) mixture was selected for both matrices. Limit of quantitation (LOQ) values were 10–25 ng/mL; linear ranges were LOQ–500 ng/ml for all analytes; correlation coefficients were greater than 0.99, and all calibrator concentrations were within 20%. Analytical recovery in blood and DBS ranged from 84.9% to 113.2% of the expected concentration for both intra- and inter-day. Analytes were stable for 1, 10, and 30 days after three freeze/thaw cycles. It was determined that the variances of the results obtained with the two matrices in the comparison study were equal for each analyte, and the results were highly correlated (*r* = 0.9625). A sensitive, accurate, and reliable chromatographic method was developed to determine amphetamine, 3,4-methylenedioxy methamphetamine, morphine, benzoylecgonine, and cannabis, by performing the same preliminary steps with whole blood and dried blood spots. It was observed that the results obtained in these two matrices were compatible and interchangeable when statistically compared.

## Introduction

Blood matrix is generally preferred due to the most evident link between an observed toxic effect and the analytical detection of drugs. Blood samples can detect the drugs under the influence of cases, provide data regarding the recent past (hours, days), offer comprehensive analytical methods, and provide a simpler pharmacological interpretation of drug effects.

Beyond being an invasive procedure, the matrix effect in whole blood due to hundreds of denatured proteins poses challenges for accurate and sensitive analysis. Thus, the search for alternative methods and techniques is an ongoing quest [1, 2].

Various techniques that utilize drying capillary blood samples have been developed to overcome the limitations imposed by blood drug analysis, and the emerging matrix has been adapted to various innovative methods. Dried blood spot (henceforth DBS) is commonly acquired by depositing small volumes (10–50 µL) of capillary blood into special paper or cards. DBS is an effective technique that provides convenience and simplicity, as it is easier to collect samples than whole blood or plasma, and a cold chain is not required to store the samples. Furthermore, being a minimally invasive technique that requires relatively low blood volume makes the DBS commonly used in various disciplines, and in addition to sample collection, transportation, and storage stages are also more manageable [3, 4].

The correlation between the analyte concentrations measured using DBS and whole blood is often discussed. Usually, the determined reference values are available only for plasma or serum samples. Therefore, a study comparing whole blood DBS and serum or plasma samples is required [5]. A good correlation between data from both DBS and whole blood methods leads to greater confidence in quantitative analysis [6]. Although the need for a small sample volume in the DBS technique is advantageous, it can pose particular difficulties in achieving the desired sensitivity.

Forensic toxicological analysis is usually carried out using chromatographic methods, in particular, high-performance liquid chromatography–tandem mass spectrometry (LC–MS-MS) technique, which provides quantitative and qualitative data for blood samples. Studies usually focused on the main parameters (column temperature, mobile phase composition, etc.) that were highlighted regarding the characteristics of the technique utilized in optimizing chromatographic methods. However, the accuracy of the method is usually dependent on the sample preparation step prior to injection into the system. These include proper handling of the sample, extraction, purification, and concentration of the targeted analytes in DBS samples [7]. In the sample preparation step, solvent extraction is frequently used in both laboratory-scale and industrial processes because of its simplicity, low cost, and suitability for thermally unstable compounds with high boiling points.

Although there are several studies developed for these drugs in dried blood samples in the literature, there is a need for a study that examines the solvent effect in detail [5, 8–15]. The type of solvent can be selected from a range of solvents and/or solvent mixtures based on their polarity matching the analytes and their suitability for further chromatographic separation step [16]. Therefore, a comprehensive list of solvents and/or mixtures was designated from the papers, including the solid-phase extraction studies’ elution step or LLE solutions, and some modifications were made [10, 13, 17–24]. This study focused on the simultaneous detection of illegal drugs which are classical and widely used in our country, including amphetamine, 3,4-methylenedioxy methamphetamine (MDMA), morphine, the cocaine metabolite benzoylecgonine, and the cannabis metabolite 11-nor-9-carboxy-tetrahydrocannabinol (Δ^9^-THC-COOH) in DBS. The DBS results were compared with whole blood, which is accepted as the gold standard method. To do so, optimum working conditions were determined using whole blood and dried blood spots, the most suitable solvent mixture was selected, and statistical evaluation was performed using authentic blood samples.

## Experimental

### Reagents and materials

(±)Amphetamine (AMP), (±)MDMA, morphine (MOR), benzoylecgonine (BZG), and (±)Δ^9^-THC-COOH in methanol at a concentration of 1 mg/mL in pure standards were purchased from Cerilliant (USA). (±) MDMA-d_5_, benzoylecgonine-d_3_, morphine-d_3_ and (±)Δ^9^-THC-COOH-d_9_ in methanol at a concentration of 100 μg/mL in pure internal standards were acquired from Cerilliant (USA).

For solvent extraction, ammonia (≥99.9% purity), ammonium formate (≥99.0% purity), hydrochloric acid (36.5%–38% purity), and deionized water were obtained from Sigma Aldrich. Acetonitrile (≥99.8% purity), ethyl acetate (≥99.8% purity), n-hexane (≥98% purity), and isopropyl alcohol (IPA) (≥99.9% purity) were supplied by Merck. Diethylether (≥99.8% purity) was obtained from Fluka, and formic acid (98%–100% purity) and sodium hydroxide (≥98% purity) were obtained from AppliChem. Chloroform (≥99.9% purity) and methanol (≥99.9% purity) were supplied by Carlo Erba. The Whatman 903 Proteinsaver Card (Sigma Aldrich) was used for DBS.

### Calibrators, quality control, and internal standards

Blank whole blood samples were taken from Ege University Faculty of Medicine Hospital Periodical Regional Blood Center. These blank samples were evaluated using the methodology detailed in this manuscript to ensure the absence of detectable amphetamine, MDMA, morphine, benzoylecgonine, and Δ^9^-THC-COOH prior to fortification with working stock solutions to prepare calibrators and quality control (QC) samples (Ethics Committee Decision No: 19-2.1T/46). Individual primary stock solutions (10 µg/mL) of amphetamine, MDMA, morphine, benzoylecgonine, and Δ^9^-THC-COOH were prepared in methanol. Working solutions from 25 to 500 ng/mL were prepared by mixing primary stock solutions and dilution in methanol.

Calibrators for all analytes (LOQs, 25, 50, 100, 250, 500 ng/mL) were daily prepared by adding working stock solutions to 50 µL blank blood. The QC samples were prepared using different lot numbers of reference standard solutions than calibrators. Five mixed QC working solutions, ranging from 25 to 500 ng/mL, were prepared in methanol. QC samples were prepared by adding working solutions to 50 µL blank blood to yield 25, 100, and 400 ng/mL amphetamine, MDMA, morphine, benzoylecgonine, and Δ^9^-THC-COOH (low, medium, and high QC, respectively). Primary stock solutions of MDMA-d_5_, morfin-d_3_, benzoylecgonine-d_3_, and Δ^9^-THC-COOH-d_9_ were diluted in methanol, to produce a mixed internal standard solution of 1000 ng/mL. All primary and working solutions were stored at −20°C in amber glass vials.

### Procedure

Specimen preparation for whole blood was achieved by creating a pool from 10 blood samples collected from the blood center after drug analysis by the routinely used in-house method. A total of 50 µL aliquots were taken from the pool and the standard solutions were added to be 100 ng/mL. The samples were treated with 1.0 mL of solvent mixture including 25 µL, 1000 ng/mL mixed internal standard solution (25 ng/mL), and vortexed for 10 s for the extraction. Then, the samples were centrifuged at 4100 rpm for 10 min, and all of the supernatant (1 mL) was transferred to a clean tube. The samples were evaporated under nitrogen gas at room temperature. In addition, 150 µL of mobile phase mixture [0.01% formic acid, 5 mM ammonium format buffer in water (Mobile phase A), and acetonitrile (Mobile phase B) 85:15 (v/v)] was added to the sediments, and the samples were transferred to an Eppendorf tube. The samples were centrifuged at 14 000 rpm for 5 min; the samples were transferred to a vial and injected into the LC–MS-MS system.

Similarly, specimen preparation for DBS was carried out by using 50 µL blood sample from the pool, and a concentration of 100 ng/mL of the standard was added. Sampling was performed on Whatman 903 filter paper using an automatic pipette. Samples were dried under atmospheric conditions for 2 h at room temperature, protected from sunlight, using a rack system to prevent interference between samples. Dried samples were punched manually with scissors, and 1 mL of solvent and/or solvent mixture, 25 µL, 1000 ng/mL internal standard mixture (25 ng/mL), was added to the dried blood spots and vortexed for 10 s. It was kept in an ultrasonic bath for 30 min at room temperature. The filter paper was removed from the Eppendorf with the help of forceps. The samples were centrifuged at 4100 rpm for 10 minutes, and the entire supernatant (1 mL) was transferred to a clean Eppendorf tube. The samples were evaporated under nitrogen at room temperature. In all, 150 µL of mobile phase mixture [0.01% formic acid, 5 mM ammonium format buffer in water (Mobile phase A), and acetonitrile (Mobile phase B) 85:15 (v/v)] was added to the residues, and the samples were transferred to Eppendorf. It was centrifuged at 14 000 rpm for 5 min, and the samples were transferred to the vial and given to the LC–MS-MS instrument.

### LC–MS-MS

The Thermo Scientific UltiMate™ 3000 UHPLC Quantum Access Max mass spectrometer was used for chromatographic separation. Analyst software version 6.80 was employed for acquisition and evaluation of data, then for performing further data analysis.

Chromatographic separation was performed on Hypersil Gold C18 (100 mm × 2.1 mm × 1.9µ) column. Gradient elution was performed with (A) 0.01% formic acid, 5 mM ammonium format buffer in water, and (B) acetonitrile at a flow rate of 0.3 mL/min. The gradient program was from 15% to 85% B over 3 min, held for 2 min, ramped from 85% to 15% B in 1 min, and re-equilibrated at 15% B for 2 min (total runtime of 7.5 min). The column oven and auto-injector sample tray were maintained at 30°C and 15°C, respectively. Mass spectrometric data were collected via positive mode electrospray ionization, the ion transitions, collision energies, polarities, and retention times are shown in Tables 1 and 2.

**Table 1.** LC–MS-MS parameters for amphetamine, MDMA, morphine, benzoilegonine, and Δ^9^-THC-COOH

Injection volume (µL)	20
Method duration (min)	7.5
Type of ion source	H-ESI
Spray voltage	Static
Positive Ion (V)	5000
Negative Ion (V)	5000
Sheath gas (Arb)	50
Aux gas (Arb)	15
Sweep gas (Arb)	1.5
Capillary temperature (°C)	350
Vaporizer temperature (°C)	0

**Table 2.** Ion transitions, collision energy, and polarity values of amphetamine, MDMA, morphine, benzoylegonine, and Δ^9^-THC-COOH

				MS transitions	
Compound	Retention time	Internal standard	Polarite	Transition	Collision energy (V)
Amphetamine	1.87	MDMA-d_5_	+	136-**91** 136-65	18, 35
MDMA	2.49	MDMA-d_5_	+	194-105 194-**135**	23, 20
Morphine	1.03	Morphine-d_3_	+	286-165 286-**201**	40, 25
Benzoylegonine	2.82	Benzoylegonine-d_3_	+	290-105 290-**168**	31, 20
Δ^9^-THC-COOH	4.57	Δ^9^-THC-COOH-d_9_	+	345-299 345-**327**	20, 15

The ions to be used for quantification are indicated in bold and underlined.

### Data analysis

The peak areas of the analytes were compared with the selection of an appropriate solvent and/or solvent mixture for whole blood and DBS samples. The peak area ratios of the analytes to the corresponding internal standards were calculated for each concentration to construct daily calibration curves via linear least-squares regression.

### Method validation

For whole blood and DBS, method validations were performed systematically following the “Standard Practices for Method Validation in Forensic Toxicology” guideline [25]. The parameters of interference studies include limit of detection (LOD), limit of quantitation (LOQ), calibration model, bias, precision, ionization suppression/enhancement, carryover, and stability.

#### Interference studies

Analyte peak identification criteria were relative retention time within ±0.15 min of the lowest calibrator and qualifier/quantifier transition peak area ratios ±20% of the mean calibrator transition ratio. The specificity of the method was assessed by analyzing 10 different drug-abstinent individuals blood specimens to evaluate potential endogenous interferences. In addition, potential interferences from commonly used drugs were evaluated by fortifying drugs into low QC sample. No interference was noted if all analytes in the low QC sample quantified within ±20% of the target concentrations with acceptable qualifier/quantifier transition ratios.

#### LOD, LOQ, and calibration model

The LOD was evaluated in quadruplicate and defined as the lowest concentration producing a peak eluting within ±0.15 min of the analytes’ retention time for the lowest calibrator, a signal-to-noise ratio of at least 3, Gaussian peak shape, and qualifier/quantifier transition peak area ratios within ±20% of the mean calibrator transition ratios. The LOQ also was evaluated in quadruplicate and defined as the concentration that met the LOD criteria, signal-to-noise ratio of at least 10, and measured concentration within ±20% of the target in four replicates. Performance at the LOQ was confirmed for each batch of specimens and was determined to have higher LOQ values than calculated.

Preliminary experiments using four sets of calibrators determined the most appropriate calibration model, by comparing goodness-of-fit for unweighted linear least squares. Calibration curves were fitted by linear least squares regression for at least five concentrations across the linear dynamic range for each analyte. In addition, calibrators were required to quantify within ±20% for the LOQ, and correlation coefficients (*R*^2^) were required to exceed 0.995.

#### Bias and precision

Intra-day and inter-day analytical recovery (bias) and imprecision were determined in five replicates at three different QC concentrations (low 25 ng/mL, medium 100 ng/mL, and high 400 ng/mL). The analytical recovery was determined by comparing the mean result of all analyses to the nominal concentration value (i.e. mean percent of expected concentration). Inter-day imprecision and analytical recovery were evaluated in three different runs with three replicates in each run, analyzed on 5 separate days. Imprecision was expressed as %RSD of the calculated concentration.

#### Ionization suppression/enhancement

In the instrumental part of the method, the postcolumn subtraction approach was chosen for ionization suppression/enhancement parameter. Two sets of samples were prepared for the experiment (low and high QC). They were not extracted, instead they were simply injected six times each. The results obtained from the datasets were calculated by calculating the percentage of ionization suppression/enhancement for target ion transitions at each concentration.

#### Carryover

Carryover was investigated in triplicate by injecting extracted blank blood samples containing internal standards immediately after samples containing target analytes at a high calibration point level (500 ng/mL).

#### Stability

Stability was evaluated using storage conditions by freeze-thaw cycles at −20°C for 1, 10, and 30 days. Blank pooled blood was fortified with analytes of interest at two concentrations (low (50 ng/mL) and high (200 ng/mL). On the analysis day, an internal standard was added to each specimen and analyzed as described for whole blood and DBS.

### Real cases and comparison samples

Whole blood samples were collected from the cases who applied to the Ege University Medical Faculty Pediatric Emergency Department with suspicion of drug use (*n* = 8). Comparison samples, on the other hand, were performed by adding a standard drug(s) to blank samples taken from the Periodical Regional Blood Center (*n* = 22). A total of 30 blood sample results were compared. The real samples and comparison sample results were analyzed with Microsoft Excel. The *t*-test: Paired Two Sample for means was performed, and the variance of whole blood and DBS was proportioned to each other. The obtained variance rate was evaluated according to the critical value of the *F*-test, and *t*-test: Two-Sample Assuming Equal Variances was applied to those with equal variances. The correlation test, which provides information about the relationship between variables and the direction and severity of this relationship, was applied, and Bland–Altman plots were also performed.

## Results

### Solvent selection for whole blood and DBS

**Figure 1.**
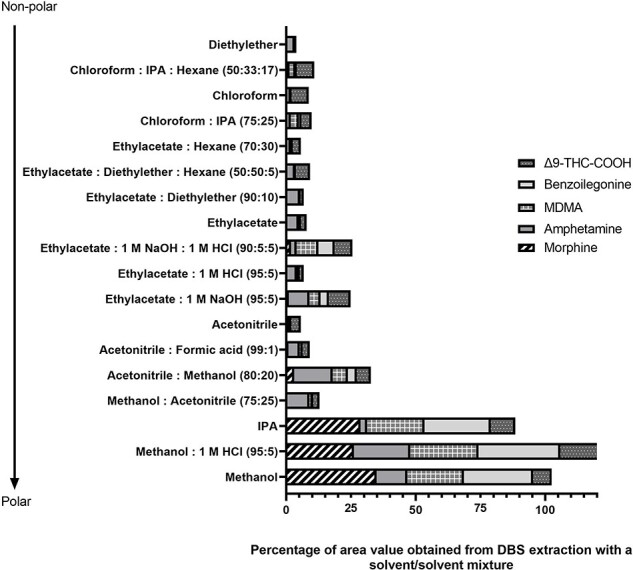
Solvent and/or solvent mixture selection in whole blood extraction.

**Figure 2.**
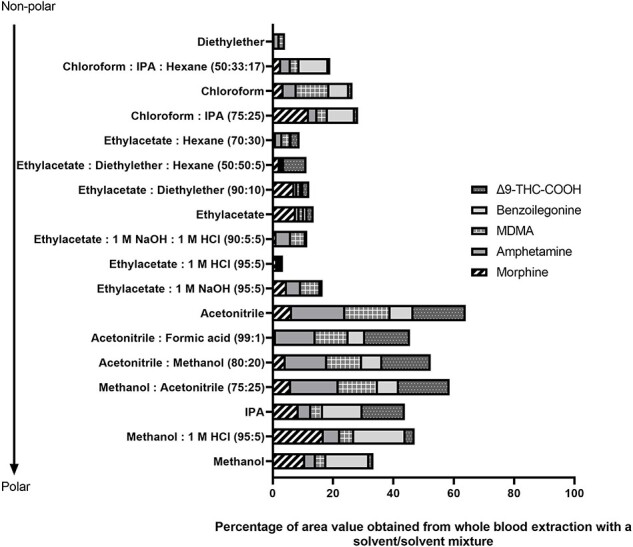
Solvent and/or solvent mixture selection in DBS extraction.

A list of solvent and/or solvent mixtures were applied to whole blood and the DBS samples spiked with targeted drugs as described in the Experimental Section and the results are presented in Figs 1 and 2, respectively. For whole blood samples, the highest abundance values for all analytes were obtained using methanol and the methanol:1 M HCl (95:5 v/v) mixture.

However, methanol and methanol:1 M HCl (95:5 v/v) mixture resulted in better abundances for MOR, BZG, and Δ^9^-THC-COOH, whereas AMP and MDMA solvents with ACN gave the best abundance values. In order to determine all analytes simultaneously and to compare results for the two biological materials, MeOH and ACN were studied with 10 unit differences in (volume:volume 100:0, 90:10, 80:20, 70:30, 60:40, 50:50, 40:60, 30:70, 20:80, 10:90, 0:100 v/v). The MeOH:ACN (40:60 v/v) ratio provided better results for all analytes.

### Interference studies

Whole blood from 10 drug-abstinent individuals did not contain interfering compounds with any peaks of interest. Multiple reaction monitoring ion chromatograms were obtained from a blank blood sample fortified with analytes at the LOQ. The AMP, MDMA, MOR, BZG, and Δ^9^-THC-COOH analytes and internal standard chromatograms are shown in Supplementary Fig. S1. Each analyte could be distinguished next to any other in the matrix environment (Supplementary Fig. S2), and the selectivity of all analytes was determined using this method in this study.

### LOD, LOQ, and calibration model

The initial experiments were conducted with four sets of calibration curves and linear least squares produced a better fit for the calibration data (data not shown). All correlation coefficients exceeded 0.995 (Table 3). LODs were between 1.6 and 7.5 ng/ml; LOQ between 10 and 25 ng/mL. The assays were linear to LOQs–500 ng/ml for all analytes.

**Table 3.** Analytical characteristics of the LC–MS-MS method for targeted analytes

		Linear range (ng/mL)	*R* ^2^	LOD (ng/mL)	LOQ (ng/mL)
Whole blood	Amphetamine	20–500	0.9989	5.0	20.0
	MDMA	25–500	0.9988	6.8	25.0
	Morphine	10–500	0.9990	2.0	10.0
	Benzoylegonine	5–500	0.9981	1.6	5.0
	Δ^9^-THC-COOH	25–500	0.9990	7.5	25.0
DBS	Amphetamine	20–500	0.9988	6.2	20.0
	MDMA	25–500	0.9972	6.8	25.0
	Morphine	20–500	0.9990	5.4	20.0
	Benzoylegonine	10–500	0.9970	2.1	10.0
	Δ^9^-THC-COOH	25–500	0.9985	7.5	25.0

### Bias and precision

The analytical recovery and imprecision were evaluated at three concentrations across the linear dynamic range. Analytical recovery in blood and DBS ranged from 84.9% to 113.2% of the expected concentration for both intra-day and inter-day analytical recovery (Table 4).

**Table 4.** Analytical recovery and imprecision data for amphetmine, MDMA, morphine, benzoylegonine, Δ^9^-THC-COOH in blood and DBS

			Analytical recovery (% of expected concentration)		Imprecision	
	Compounds	Expected concentration (ng/mL)	Intra-day, (*N* = 5) Mean ± SD	Inter-day, (*N* = 3) Mean ± SD	Intra-day (%RSD)	Inter-day (%RSD)
Whole blood	Amphetamine	25	96.0 ± 9.0	84.9 ± 1.3	9.4	1.5
		100	100.6 ± 6.4	93.5 ± 2.0	6.3	2.2
		400	98.4 ± 3.5	99.6 ± 2.6	3.5	2.6
	MDMA	25	88.5 ± 8.8	94.5 ± 14.9	9.9	15.8
		100	101.2 ± 6.4	102.7 ± 9.9	6.3	9.6
		400	95.5 ± 7.1	97.5 ± 5.0	7.4	5.1
	Morphine	25	97.8 ± 11.2	87.9 ± 8.4	11.4	9.5
		100	100.0 ± 11.6	95.5 ± 5.4	11.6	5.7
		400	98.4 ± 5.5	98.3 ± 3.7	5.6	3.7
	Benzoylegonine	25	102.2 ± 10.8	110.4 ± 10.3	10.7	9.3
		100	100.0 ± 7.5	96.9 ± 7.8	7.5	8.1
		400	100.4 ± 7.0	98.0 ± 5.2	7.0	5.3
	Δ^9^-ΤΗC-CΟΟΗ	25	113.2 ± 12.5	84.9 ± 1.3	11.1	1.5
		100	108.8 ± 9.6	93.5 ± 2.0	8.9	2.2
		400	98.4 ± 3.5	99.6 ± 2.6	3.5	2.6
DBS	Amphetamine	25	98.2 ± 11.0	89.0 ± 12.9	11.2	14.5
		100	104.8 ± 7.9	100.4 ± 16.8	7.6	16.7
		400	98.1 ± 7.0	92.5 ± 7.9	7.2	8.6
	MDMA	25	92.8 ± 5.6	107.5 ± 12.0	6.0	11.2
		100	98.9 ± 4.8	101.0 ± 2.6	4.8	2.6
		400	96.1 ± 9.0	96.2 ± 7.4	9.4	7.7
	Morphine	25	90.0 ± 6.3	87.3 ± 7.3	7.0	8.3
		100	102.4 ± 6.4	99.6 ± 7.3	6.2	7.4
		400	98.7 ± 9.2	100.8 ± 8.6	9.4	8.6
	Benzoylegonine	25	107.2 ± 4.5	107.3 ± 8.5	4.2	8.0
		100	95.5 ± 12.1	88.4 ± 5.2	12.7	5.8
		400	100.7 ± 12.4	100.9 ± 10.1	12.3	10.0
	Δ^9^-ΤΗC-CΟΟΗ	25	103.7 ± 9.2	113.5 ± 145.6	8.8	13.8
		100	91.7 ± 24.5	99.6 ± 9.3	26.7	9.3
		400	96.7 ± 4.5	96.6 ± 4.5	4.6	4.6

### Ionization suppression/enhancement

They calculated the % suppression/enhancement for 25 and 400 ng/mL and for the internal standard in both sets. The results suggested suppression of 1.2% and 12.7% for amphetamine, 9.5% and 18.7% for MDMA, 3.8% and 20.8% for morphine, 21.8% and 8.5% for benzoylecgonine and 2.1% and 23.7% for Δ^9^-THC-COOH at 25 and 400 ng/mL concentrations in whole blood, respectively.

Also, results suggested suppression of 5.1% and 21.9% for amphetamine, 14.3% and 2.3% for MDMA, 6.1% and 16.6% for morphine, 10.3% and 0.3% for benzoylecgonine and 4.3% and 11.8% for Δ^9^-THC-COOH at 25 and 400 ng/mL concentrations in DBS samples.

For both matrix ionization suppression was less than 25%.

### Stability and carryover

**Figure 3.**
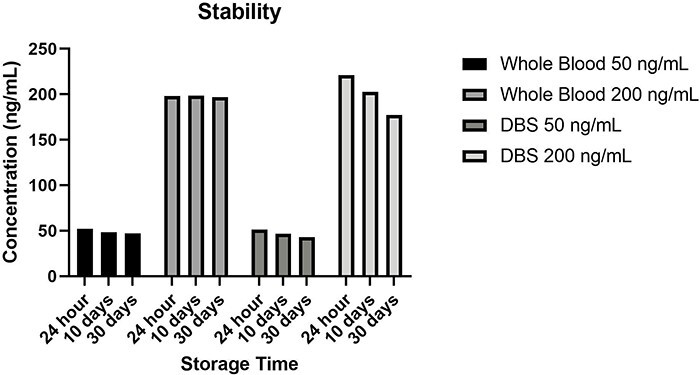
Whole blood and DBS stability at low (50 ng/mL) and high concentration (200 ng/mL).

Analytes at two concentrations in blood samples were stable at −20°C temperature for 1, 10, and 30 days after three freeze-thaw cycles (Fig. 3). The mean percent analyte loss for all analytes was 6.5%.

After working on the 500 ng/mL target analytes sample, it was determined that there were low amounts of analytes. The carryover in the syringe revealed that washing the syringe with acetonitrile before and after injection was necessary to remove residual analytes. After the syringe was washed with acetonitrile, no evidence of analyte carryover was observed. None of the negative specimens injected after samples containing twice the upper limit of linearity contained an analyte satisfying the assay LOQ criteria (*n* = 3).

### Real cases and comparison samples

The suitability of the interchangeability of whole blood and DBS matrices was analyzed statistically, and comparison study results were used (*n* = 30). Results for five analytes were evaluated in the study (*n *= 30 × 5 = 150). Of the results, 98 were negative and 52 were positive. Positive results were evaluated in the comparison study. According to the *F*-test, the variances of the results obtained by the two techniques for each analyte were equal (Table 5).

**Table 5.** *F*-test, *t*-test, and correlation values for positive drug results for whole blood and DBS samples

	*F*-test			*t*-Test: Two-Sample assuming equal variances			Pearson correlation
Targeted analyte	Variance ratio	Degrees of freedom	p value	Critical	p value		Coefficient value
Amphetamine	1.189	8	3.438	1.745	0.007	p < p_critical_	0.986
MDMA	1.087	7	3.787	1.745	0.100	p < p_critical_	0.998
Morphine	1.080	7	3.787	1.745	0.079	p < p_critical_	0.999
Benzoylegonine	0.741	8	3.438	1.734	0.115	p < p_critical_	0.990
Δ^9^-ΤΗCCΟΟΗ	0.649	13	2.533	1.701	0.437	p < p_critical_	0.963

**Figure 4.**
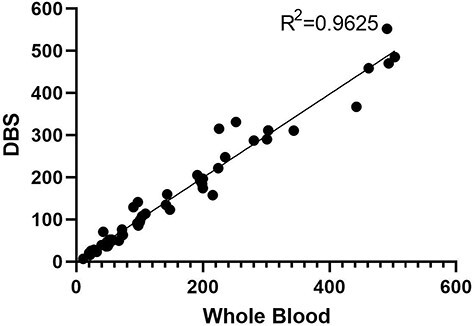
Data from a method comparison experiment with the DBS plotted against the whole blood for all positive drugs with Bland–Altman plot.

The correlation coefficient was calculated as *r* = 0.9625 for whole blood and DBS samples [the correlation was significant at the 0.01 level (two-tailed)]. If this value is positive and close to 1; it can be said that as one variable increases, the other increases, and there is a strong positive correlation (Fig. 4).

## Discussion

Cannabis is the most widely abused substance in Turkiye and worldwide [26, 27]. The primary tetrahydrocannabinol metabolite, Δ^9^-THC-COOH, is highly variable in blood values, depending on the frequency of consumption and the time difference between drug intake and blood collection. ∆^9^-THC-COOH concentrations in the blood can be used to differentiate between occasional and regular cannabis use because of the long plasma half-life of ∆^9^-THC-COOH and its accumulation after frequent cannabis use. The concentrations of free Δ^9^-THC-COOH and/or glucuronide in blood have been suggested as markers for frequent THC exposure [28]. Fabritius *et al*. used a threshold concentration of 40 ng/mL for free Δ^9^-THC-COOH in whole blood. An experimental study was conducted to determine the threshold value for heavy and occasional use [29]. Although THC and its metabolites, which behave differently from other analytes, were not included in many DBS studies, in this study, the Δ^9^-THC-COOH limit of quantification value in blood and DBS was determined as 50 ng/mL and could be determined simultaneously with other analytes. As a report, other commonly abused drugs are amphetamine derivatives, morphine, and cocaine [30]. The analysis of these drugs in blood and in DBS, which creates more stable structures, is very important in the field of forensic toxicology.

In a review study by Maurer H. on the analysis of analytes, which are essential in forensic and clinical toxicology, in whole blood, serum, and plasma samples using chromatographic systems, and in the chapter of the book compiled by Ertaş H. *et al*., preliminary preparation methods, used mobile phases, columns, and ionization types are presented in tables [31, 32]. In our study, we compared the LLE extraction solvents for whole blood and DBS samples by considering and evaluating related literature. It was reported that water and a water–methanol mixture (10:1 v/v) was used as the LLE solvent in one study [33]. In the trials conducted within the scope of this study, it was observed that the water, water: methanol mixture only diluted the blood samples, and there was no precipitation or separation. In the DBS samples, it was observed that the entire dried matrix was dissolved, and a clearer but still red-pink solution was formed compared with blood. These solvents are not included in Figs 1 and 2, as the samples contain particles and pollution that are too dense, making them unsuitable for the chromatography device being injected directly. The responses of the five analytes to different solvents are different, and Δ^9^-THC-COOH, which was not included in many studies, was the most remarkable factor in this study.

During establishing the optimum solvent selection for LLE for these analytes, the study continued under conditions in which the relative absorbance values were obtained across analytes. While 100% acetonitrile delivered the highest area value for AMP, MDMA, and Δ^9^-THC-COOH in the blood samples, methanol:1 M HCl (100:10 v/v) provided the highest results for MOR and BZG. On the other hand, in DBS samples, mixtures containing methanol presented much higher area values for MOR, BZG, and Δ^9^-THC-COOH, while ethyl acetate solvents with acid and basic solvent-added presented the best area values for AMP and MDMA. Our study procedure was updated to create the same working conditions for blood and DBS, and a study comparing methanol and acetonitrile was designed. As a result, a methanol–acetonitrile (40:60 v/v) ratio was selected for both matrices. This ratio gave the better results for all analytes, especially Δ^9^-THC-COOH was decisive at this stage.

DBS is one of the best-known alternatives to whole blood sampling for the detection of an analyte. The DBS technique’s most important advantage is the minimal blood volume required to test an analyte [34]. However, in studies using a limited sample, the amount of drug in the sample is diluted during the extraction phase. Therefore, it may be concentrated and diluted in the processes being performed. This study started with 50 µL of the blood sample, and the final volume was given to the device as 150 µL. In the analyses performed in LC–MS-MS, when a 50 µL injection volume was applied, it did not enable the same sample to be analyzed repeatedly. Repeated analysis was possible by reducing the injection volume to 20 µL. Repeated analysis, calculating the mean value of a sample, and using this value can aid avoid problems such as injection repeatability, that is, whether the sample was prepared cleanly.

In line with the simultaneous determination of the studied analytes, it was determined that there was a decrease in the 30-day freeze/thaw cycle stability study of Δ^9^-THC-COOH in our study (recovery value was calculated 44.6%). This problem can be avoided by storing the samples at lower temperatures or using different drying techniques, which will likely allow different studies to be applied in the future.

In addition, method development was conducted with blood samples and samples taken from the blood center for DBS validation. One limitation of this study was that hematocrit levels were not examined separately. To prevent differences in hematocrit levels in the samples, a blood pool was created. The blood pool was created with at least 10 different blood samples to ensure that the blood samples were from different blood groups. During the actual sample collection stage, whole blood samples were collected.

Centre punch and whole spot manual punching and automatic punching performed on drying samples to prepare DBS samples [19,35]. A study comparing these two techniques stated that these two methods could be used interchangeably with a correction factor [35]. In this study, all of the dried blood spots formed were cut with scissors (manually punching), and the entire sample was used for extraction, which is another limitation of our study.

Numerous methods have been published for the qualitative and quantitative analysis of abused drugs in the blood and other body fluids. One of them, a study conducted in Norway in 2011, analyzed 28 drugs in whole blood. Øiestad *et al*. repoted that, the solid phase extraction (SPE) method was used, yielding results in the range of 82%–87% for THC, 94%–114% for morphine, 106%–131% for MDMA, 96%–115% for amphetamine, and 92%–111% for cocaine [36]. In our study, blood samples were extracted with LLE, and recovery ranges were 94%–100% for morphine, 94%–100% for amphetamine, 88%–101% for MDMA, 99%–102% for benzoylecgonine, and 101%–113% for Δ^9^-THC-COOH. In the recovery range, results were obtained within the desired ±20% recovery range for biological samples. Øiestad *et al*. repoted that LOQ values, important in instrument sensitivity, were lower for all analytes than in this study. This may be due to preliminary preparation with the SPE technique using a 500 µL blood sample.

It was determined that our results were compatible with the study conducted by Simões *et al*. They use a methanol:acetonitrile (3:1 v/v) ratio for the LLE solvent for the preparation of DBS samples, and the subsequent processing steps were in significant agreement with our study. In their study, the stability of the samples was run at regular intervals for up to 240 days. The calibration curve linearity was extended to 500 ng/mL as in our study. The LOQ values of the study ranged from 1 to 5 ng/mL for MOR, benzoylecgonine, and amphetamine and its derivatives. Recovery values for low (20 ng/mL) and high (200 ng/mL) concetrations have been reported as 61%–51% for morphine, 80%–85% for amphetamine, 84%–86% for MDMA, and 91%–89% for benzoylecgonine [10]. Ambach *et al*. developed a fast and sensitive DBS method for detecting 64 newly designed psychoactive drugs. Because of the long list of amphetamine derivatives, cathinones, and tryptamine derivatives, it was reported that the DBS technique was more stable for some compounds. They reported that this technique is suitable for screening purposes [13]. In the DBS study conducted by Kacargil *et al*. in 2020, amphetamine derivatives, morphine, codeine, cocaine, and benzoylecgonine were analyzed, and the ethylacetate:methanol couple was used as the LLE solvent. The developed method was applied to 59 cases, but the results of these cases were not statistically evaluated [14]. However, in these studies, the most abused substance cannabis, and its metabolites were not examined.

In one of the studies on the correlation between analyte concentrations measured with DBS and whole blood, Jantos *et al*. investigated MDMA and its main metabolite, 3,4-methylenedioxy amphetamine (MDA). The mean concentrations of the results obtained from whole blood and DBS samples from actual samples were tested with the *t*-test to test the inequality hypothesis. Statistical analyses showed no significant difference in MDMA or MDA levels at the end of the methods [20]. In a comparison study by Boy *et al*., morphine and 6-monoacetylmorphine (6-AM) were analyzed in DBS and whole blood materials. The parameters of the DBS technique were determined using experimental design techniques. The DBS matrix stabilized 6-AM quite well; using direct extraction from blood and DBS into real samples revealed no significant difference between the two methods (Boy *et al*., 2008). In our study comparing variance ratios with the critical values, we found that the results obtained for all analytes had equal variance. For example, there was no difference between the two matrices after the t-test: Two-Sample Assuming Equal Variances test. In addition, as shown in Fig. 4, the results were correlated with the correlation test results and were compatible with the Bland–Altman plot.

It is shown that there are also some disadvantages to DBS. Some of these materials are nonuniform, effective drying for uneven distribution of blood droplets on a porous substrate due to differences in blood hematocrit volumes among of different persons, formation of clots or spots during bloodstain formation, heterogeneous distribution of blood analytes on porous paper due to the effect of thin-layer chromatography, and bloodstain preservation. The absence of the method can be described as moisture, contamination, and analyte degradation. These limitations result in a variable and sometimes high failure rate in producing a perfect stain with the DBS technique. However, it was reported that such failures in forming a perfectly dried bloodstain could be avoided by confining the porous materials into the micro/nanostructure (Alsous *et al*., 2020; Kumar *et al*., 2019).

## Conclusion

A sensitive, accurate, and reliable chromatographic method was developed to determine amphetamine, MDMA, morphine, benzoylecgonine and especially Δ^9^-THC-COOH by performing the same preliminary steps as whole blood and dried blood spots. Two comparable methods were validated, using the same extraction solvent mixture, using a 50 µL sample volume in both matrices. The results suggest that the analytes can be stored under suitable conditions for up to 30 days. Finally, we observed that the results obtained in these two matrices were compatible and interchangeable when statistically compared.

## Supplementary Material

bkae081_Supp

## Data Availability

The data underlying this article will be shared on reasonable request to the corresponding author.
