# Supplementary material for: Liquid–liquid extraction solvent selection for comparing illegal drugs in whole blood and dried blood spot with LC–MS–MS
Source: J Anal Toxicol. 2024 Oct 5;49(1):26–35. doi: 10.1093/jat/bkae081 (PMC11753396; doi:10.1093/jat/bkae081)
Supplement: bkae081_Supp [file bkae081_supp.zip › jat-24-4140-File008.docx]

**Supplementary**


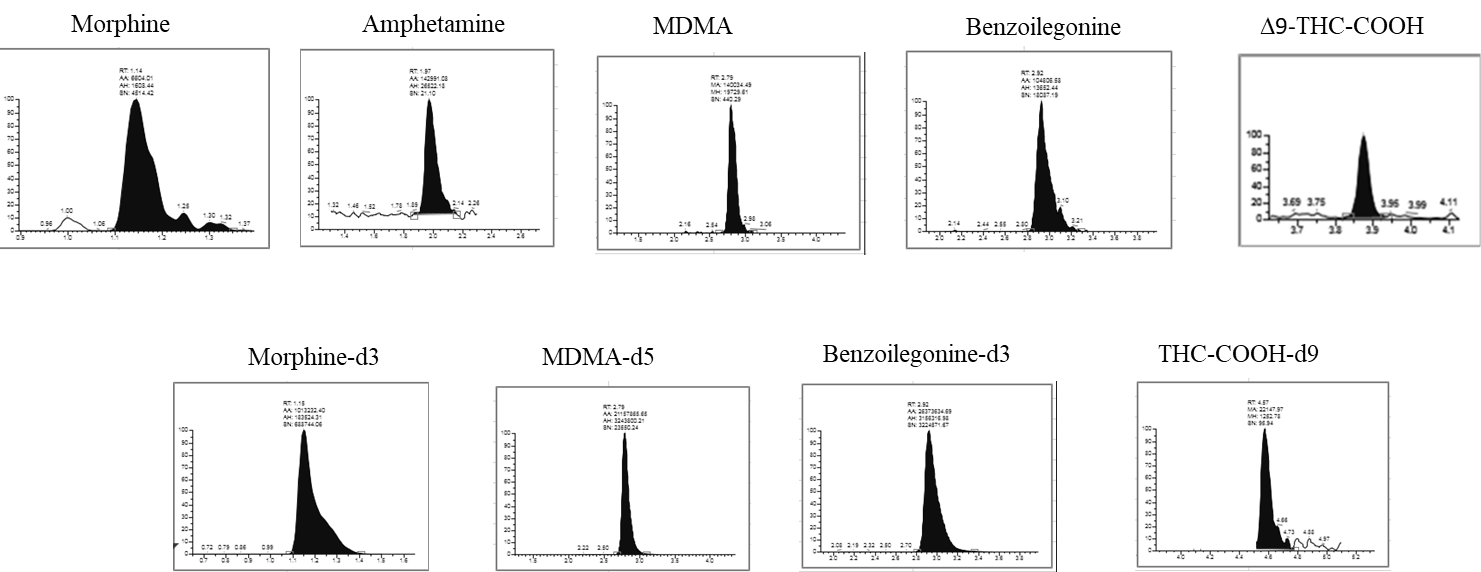


**Supplementary Figure 1.** Chromatograms obtained for a whole blood sample spiked with 25 ng/ml of mixed standard solution and internal standard

**
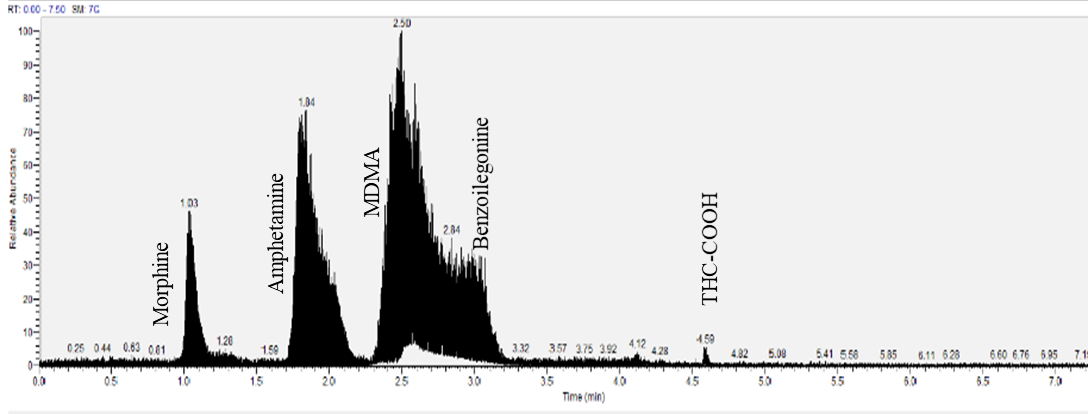
**

**Supplementary Figure 2.** Chromatogram containing 200 ng/mL analytes standard mix in DBS samples
